# Recent Advances of Bioresponsive Nano-Sized Contrast Agents for Ultra-High-Field Magnetic Resonance Imaging

**DOI:** 10.3389/fchem.2020.00203

**Published:** 2020-03-20

**Authors:** Hailong Hu

**Affiliations:** ^1^School of Aeronautics and Astronautics, Central South University, Changsha, China; ^2^Research Center in Intelligent Thermal Structures for Aerospace, Central South University, Changsha, China

**Keywords:** nano-sized, contrast agents, magnetic resonance imaging, multimodal imaging, artificial intelligence

## Abstract

The ultra-high-field magnetic resonance imaging (MRI) nowadays has been receiving enormous attention in both biomaterial research and clinical diagnosis. MRI contrast agents are generally comprising of T_1_-weighted and T_2_-weighted contrast agent types, where T_1_-weighted contrast agents show positive contrast enhancement with brighter images by decreasing the proton's longitudinal relaxation times and T_2_-weighted contrast agents show negative contrast enhancement with darker images by decreasing the proton's transverse relaxation times. To meet the incredible demand of MRI, ultra-high-field T_2_ MRI is gradually attracting the attention of research and medical needs owing to its high resolution and high accuracy for detection. It is anticipated that high field MRI contrast agents can achieve high performance in MRI imaging, where parameters of chemical composition, molecular structure and size of varied contrast agents show contrasted influence in each specific diagnostic test. This review firstly presents the recent advances of nanoparticle contrast agents for MRI. Moreover, multimodal molecular imaging with MRI for better monitoring is discussed during biological process. To fasten the process of developing better contrast agents, deep learning of artificial intelligent (AI) can be well-integrated into optimizing the crucial parameters of nanoparticle contrast agents and achieving high resolution MRI prior to the clinical applications. Finally, prospects and challenges are summarized.

## Introduction

As one of the most attractive and useful techniques for non-invasive imaging, magnetic resonance imaging (MRI) shows its great superiority in the practical application of clinic diagnosis, as well as the biomedical research (Zhao et al., 2001, 2013, 2015; Werner et al., 2008; Kim et al., 2009; Lee et al., 2012; Bao et al., 2018; Pellico et al., 2019; Liu et al., 2020). It is well-acknowledged that spatial resolution can be promoted by high magnetic field over 3 T, demonstrating a high signal-to-noise ratio (Vaughan et al., 2009; Rosenberg et al., 2010; Zhou et al., 2015b; Ni et al., 2016; Zhang et al., 2016). This result is usually evidenced by the MRI study on small animals under the applied high field (over 7 T) (Nakada, 2007; Werner et al., 2008; Faucher et al., 2012; Ni et al., 2016). Moreover, compared with 1.5 or 3.0 T MRI, ultra-high-field MRI shows its unique advantage in medical imaging, especially in the field neuroscience, where functional brain responses can be non-invasively measured with increasing sensitivity and greater spatial resolution under ultra-high-field MRI (de Martino et al., 2018). However, much more effort should be devoted to exploring the ultra-high-field MRI to eventually achieve the enhanced solution and sensitivity for clinical imaging diagnosis (Duyn, 2012; Zhao et al., 2013; Chang et al., 2016; Gautam et al., 2019; Harris et al., 2019; Rajamanickam, 2019).

**Figure 1:**
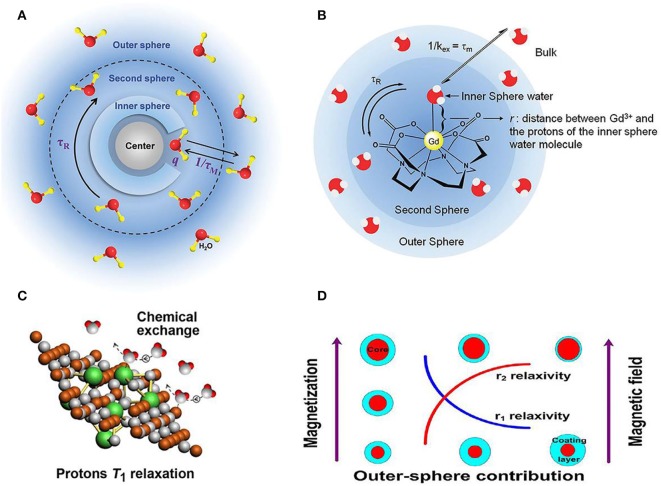
Relationship between structure and relaxivity of nanoparticle contrast agent for MRI. **(A)** Schematic diagram of mechanism for contrast agents (Ni et al., 2017); Copyright 2017, reproduced with permission from The Royal Society of Chemistry **(B)** factors illustrated for affecting contrast agent's relaxivity (Verwilst et al., 2015); Copyright 2015, reproduced with permission from The Royal Society of Chemistry. **(C)** Efficient chemical exchange for protons to accelerate T_1_ relaxation (Zhou et al., 2015a); Copyright 2015, reproduced with permission from American Chemical Society **(D)** surface modification of nanoparticles contributing to MRI T_2_ contrast agents (Zhang et al., 2016); Copyright 2016, reproduced with permission from American Chemical Society.

The lanthanide ions such as Dy^3+^ and Ho^3+^ contrast agent ions are generally used for high magnetic field MRI (Das et al., 2011, 2012; Harris et al., 2016; Ni et al., 2016; Zhang et al., 2016). Signal intensity of MRI is affected by the relaxation rate of *in vivo* water protons. By using the contrast agent with varied contents, the MRI signal intensity can be changed, where paramagnetic metal ion in contrast agent with different concentrations will positively alter the relaxation rate of nearby water proton spins (Idisi et al., 2019; Kubíčková et al., 2019). The contrast agent for MRI has been illustrated in Figure 1, where several factors affecting the MRI have been clearly indicated with the listed examples. Figure 1A shows the general mechanism of contrast agent, where the change of hydrogen atom's magnetization in water plays a predominant role in deciding the capability of generated contrast for contrast agent. For the atomic structure of paramagnetic contrast agent, paramagnetic center, inner sphere, secondary sphere and outer sphere are included. The relaxation contribution is quantitatively determined by the location between water protons and contrast agent. Water molecules coordinated to paramagnetic center will contribute to the inner sphere relaxation contribution and bulk water molecules will be responsible for the outer sphere relaxation (Ni et al., 2017; Marasini et al., 2020).

Moreover, take the gadolinium ions based contrast agent as another case, surface modification of organic ligands will be tightly binding to gadolinium ion avoid the toxicity for clinical diagnosis (Csajbok et al., 2005; Bridot et al., 2007). For this case, the hydrogen are bonded to the ligand instead of water molecules bounding to gadolinium ion, forming the second sphere in Figure 1B (Verwilst et al., 2015). Other crucial factors to the relaxation mechanism of contrast agent, such as chemical structure and surface modification (Zhu et al., 2016; Wang et al., 2018; Jin et al., 2019; Yin et al., 2019; Zhang et al., 2019) are also illustrated (Figures 1C,D), where efficient chemical exchange for protons accelerates relaxation and surface modification of nanoparticles contributes to MRI T_2_ contrast agents, respectively (Zhou et al., 2015a; Zhang et al., 2016). Specifically, the effect of particle size and magnetic field on influencing relaxivities of r_1_ and r_2_ has been investigated based on the out-sphere theory, showing the increased r_2_ contributed by the increasing core size, decreasing coating layer thickness or increasing magnetic field.

When employing contrast agents for MRI, contrast agent shall possess the following distinct features for better imaging (Zhou et al., 2019): (a) Nano-size, achieve the maximum recognition; (b) diagnosis with high accuracy; (c) Compatibility, non-cytotoxic and biocompatible; (d) Stability, both chemical and photochemical stable; (e) Metabolism; renal excretion from body (Qin et al., 2007; Huang et al., 2010; Botar et al., 2020).

In addition, the recent progress of emerging artificial intelligence (AI) has advanced the exploration of ultra-high-field MRI, where AI in conjunction with MRI is supposed to be prevalently used in many cases, ranging from imaging reconstruction to the final clinical decision support (Busch, 2019). For instance, Sheth et al. discussed the AI in interpreting breast cancer on MRI, aiming to enhance the efficacy and accuracy of diagnosis (Sheth and Giger, 2019). To bridge the current gap between virtual reality and neuropathology, AI and ultra-high-field MRI with enhanced resolution will inevitably advance the knowledge of microstructure changes in varied pathogenetic stages (O'sullivan et al., 2019).

## Recent Advances of Contrast Agents for Ultra-High-Field Mri

To reveal the efficacy of a variety of contrast agents, crucial parameters have been defined to indicate the efficiency and species of contrast agents. Transverse relaxivity (r_2_), longitudinal relaxivity (r_1_) and the ratio of r_2_/r_1_ are used to evaluate the contrast efficiency of MRI contrast agent, where a high ratio of r_2_/r_1_ generally results in a high contrast efficiency (Shen et al., 2017b). A more efficient T_2_ contrast agent instead of T_1_ contrast agent will be determined by the increasing ratio of r_2_/r_1_ (Das et al., 2012). Metal ion includes the electron orbital motion and electron spin motion. The electron spin magnetic moment plays a predominant role in determining the longitudinal water relaxation (r_1_). The existence of electron spin angular momentum of adjacent ions contributes to an enlarged total electron angular momentum, leading to a high r_1_. On the contrary, total electron angular momentum will be quite small if contribution is only from electron orbital angular momentum.

Gadolinium chelates are commonly used as contrast agents owing to its advantages of offering superior non-invasive visualization for ailments (Rogosnitzky and Branch, 2016; Marangoni et al., 2017; Rees et al., 2018; Clough et al., 2019). However, both the short circulation lifetime in the body and the relatively low proton relaxation efficiency substantially limit its wide application. Moreover, to achieve the high proton relaxation efficiency, high concentration of contrast agents is required, which will deteriorate human's body in terms of the side effect induced by Gd^3+^ ions.

Alternatively, nanoparticle with magnetic responsive atom is becoming one of the most promising candidates to be used as contrast agents, such as transition metal ions (Cu^2+^, Fe^2+^/Fe^3+^,Co^2+^, and Mn^2+^, lanthanide metal ions (Eu^3+^, Gd^3+^, Ho^3+^, and Dy^3+^) (Tromsdorf et al., 2007; Mahmoudi et al., 2011; Busquets et al., 2015; Ni et al., 2017; Sousa et al., 2017; Guo et al., 2019; Hai et al., 2019; Wahsner et al., 2019; Botar et al., 2020). Furthermore, iron-based nano-system is highly recommended as spin-spin imaging or imaging probe because of its merits of electronic, magnetic, optical properties at nano scale and the excellent *in vivo* stability as well (Li et al., 2013). On the other hand, high surface area of nanoparticle promotes the chemical reactivity and make it viable to be modified with the biological and bioactive surfactants. Therefore, owing to these superior benefits, nanoparticles have been regarded as one of the promising alternatives for imaging contrast agents in future (Bobo et al., 2016; Chen et al., 2016; Shen et al., 2017c).

**Figure 2:**
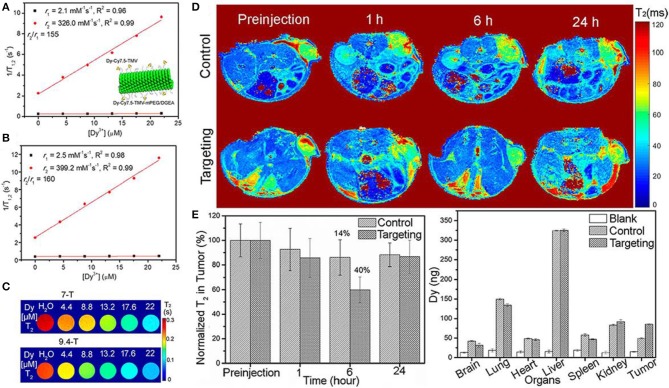
Plots of water proton relaxation times of longitudinal r_1_ and transversal r_2_ as a function of Dy^3+^ content (Dy-Cy7.5-TMV-mPEG) under magnetic field of **(A)** 7 T (The insert shows the schematic diagram of modified Dy-Cy7.5-TMV-mPEG/DGEA nanoparticles); **(B)** 9 T; **(C)** T_2_ mapping phantoms at different contents of Dy^3+^ under magnetic field of 7 T and 9.4 T; **(D)** *in vivo* MRI mapping as a function of time after intravenous injection of control and targeting group Dy-Cy7.5-TMV-mPEG/DGEA; **(E)** Analysis of T_2_ as a function of time and distribution of Dy^3+^ in organs after 24 h injection (Hu et al., 2017); Copyright 2017, reproduced with permission from American Chemical Society.

It is well-recognized that ultra-high-field MRI has achieved the increasing prevalence in both fundamental research and clinical applications (Dyke et al., 2017; Huelnhagen et al., 2017; Lehericy et al., 2017; Hametner et al., 2018). A bimodal contrast agent of Dy^3+^(DOTA)/Cy7.5-conjugated tobacco mosaic virus (TMV) was developed for ultra-high-field MRI, confirming its high transverse relaxivity r_2_ and suitable for both NIRF imaging and T_2_ MRI in cancel cells diagnosis (Hu et al., 2017). Figures 2A,B show the relaxivity of contrast agent under ultra-high-magnetic of 7.0 and 9.4 T. Both high r_2_ and high ratio of r_2_/r_1_ are obtained, showing the high efficiency of T_2_ contrast agent for ultra-high-field MRI application. To further confirm the feasibility of this contrast agent, experiments are performed on concentration dependent phantom images (T_2_-mapping) of contrast agent in water solutions at 7.0 and 9.4 T, showing a pronounced negative contrast gradient as a function of contrast agent concentration (Figure 2C). Till now, this kind of contrast agent demonstrates its great potential for ultra-high-field MRI. Furthermore, this contrast agent is to target tumors of mouse models by *in vivo* T_2_-mapping MRI to check its ability. The impact of targeted and untargeted nanoparticles on local tissues is determined based on the relaxation times. Figure 2D shows a much stronger signal enhancement after contrast agent postinjection for a certain of hours (6 h) and the recovery is achieved after T_2_ relaxation of 24 h. The biodistribution of nanoparticle contrast agent in organs and tumors are also determined (Figure 2E), showing more targeted nanoparticles accumulated in the tumors of mice than that of control nanoparticles, which is well-consistent with *in vivo* MRI images. Moreover, a certain number of nanoparticles are expected to stay in the liver owing to circulation of mononuclear phagocytic system. Consequently, the *in vivo* MRI results of nanoparticle confirm its efficiency as contrast agent.

**Figure 3:**
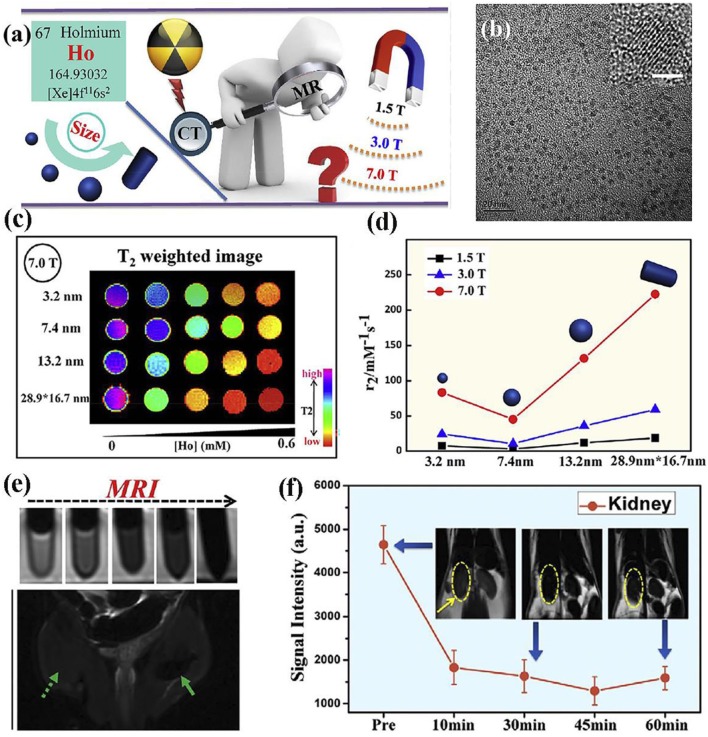
**(a)** Schematic diagram of nanoparticle for high field MRI; **(b)** TEM image of nanoparticle (The scale bar of inset image is 2 nm); **(c)** nanoparticles with varied size at 7.0 T; **(d)** transverse relaxivity r_2_ of nanoparticles with varied size at magnetic fields of 1.5, 3.0, and 7.0 T, respectively; **(e)** MRI of nanoparticles at different contents; **(f)** *in vivo* ultra-high-field MRI of kidney form mice after the intravenous injection of nanoparticles (Ni et al., 2016); Copyright 2016, reproduced with permission from Elsevier.

Ni et al. prepared the NaHoF_4_ nanoparticles via surface modification and studied the size effect of nanoparticles on MRI contrast agent performance under varied magnetic fields (Figure 3) (Ni et al., 2016, 2017). The optimal size of nanoparticles has been eventually achieved to test ultra-high-field MRI, showing an excellent biocompatibility and great promising candidate for future high field MRI. Moreover, as the size change of nanoparticles will bring alterable performance to MRI, whose detailed mechanism are also investigated, demonstrating the predominant curie mechanism for size below 7 nm and the main contribution of dipolar mechanism for size over 7 nm. Both rod-like NaDyF_4_ and NaHoF_4_ nanoparticles were prepared via a high temperature synthesis process and they are the promising candidate as T_2_ contrast agents under the high field of 9.4 T MRI. Simulation results indicate the increase of 100 times for relaxivity when magnetic field increases from 1 to 10 T. Parameter such as the size was also studied and showed an increase of 30 times for both r_1_ and r_2_ when nanoparticle core size increases from 5 to 15 nm (Zhang et al., 2016).

**Figure 4:**
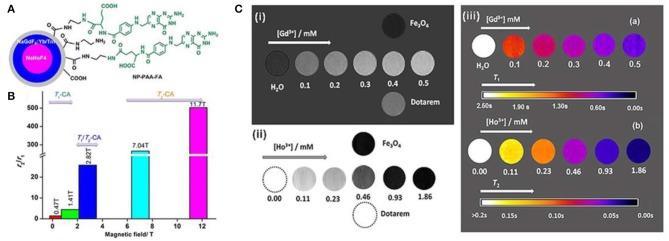
**(A)** Atomic structure of prepared NP-PAA-FA; **(B)** the varied r_2_/r_1_ as a function of magnetic field; **(C)** (i) shows the T_1_-weighted MRI; (ii) shows the T_2_-weighted MRI; (iii) shows the maps of solution with NP-PAA-FA at magnetic field of 3.0 T (Biju et al., 2018); Copyright 2018, reproduced with permission from John Wiley and Sons.

Except the contrast agent developed for T_2_-weighted MRI, the attempts into developing dual-mode T_1_/T_2_ contrast agents are also reported. Biju et al. reported a new type of contrast agent which can be used in either ultra-high magnetic field or multimodal imaging of MRI and optical imaging (Biju et al., 2018). This kind of contrast agent NP-PAA-FA is designed with core-shell structure with an average size of about 24 nm, and surface modification/functionalization is applied to enhance the contrast agent's biocompatibility. Both MRI and proton nuclear magnetic relaxation dispersion studies confirm the contrast agent's feasibility of behaving as a dual-weighted contrast agent at 3 T, and acting as a highly efficient T_2_ weighted MRI contrast agent. Figure 4A shows the core-shell structure of prepared NP-PAA-FA contrast agent, and the obtained high values of ratio r_2_/r_1_ at ultra-high- field indicate its ability for MRI contrast agent (Figure 4B). To confirm the dual-modal character of NP-PAA-FA contrast agent for MRI, phantom images of concentration dependent of NP-PAA-FA water solution are compared to that commercially available Dotarem or Fe_3_O_4_ contrast agent, showing the pronounced alterable contrast in both T_1_ and T_2_ images as a function of concentration (Figure 4C). Therefore, great potential of NP-PAA-FA as a multimodal constant agent has been demonstrated, which will pave the way for studying more, novel and suitable contrast agent for ultra-high-field MRI.

**Figure 5:**
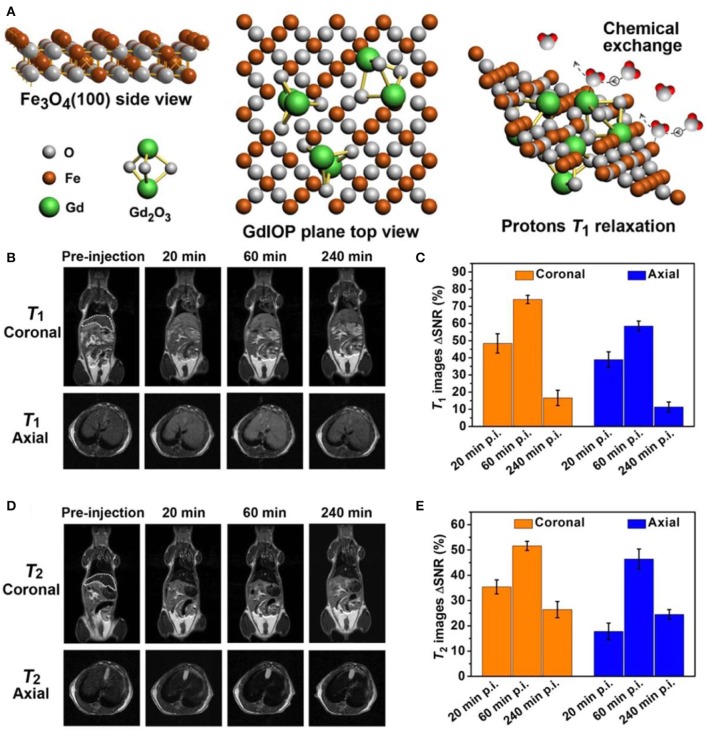
T_1_-T_2_ dual-modal MRI at ultra-high-field of 7.0 T: **(A)** Atomic structure of GdIOPs, including the top view of Fe_3_O_4_, plane to view of GdIOP and chemical exchange in promoting protons T_1_ relaxation. **(B–E)** GdIOPs acting as T_1_-T_2_ dual-modal MRI contrast agents (Zhou et al., 2015a); Copyright 2015, reproduced with permission from American Chemical Society.

Despite the efficient of surface modification technique to improve the biocompatibility, nanobiointerface in conjunction with surface structure engineering provides another possibility to develop high performance dual-modal MRI contrast agent (Zhou et al., 2015a). Small molecules of sodium citrate and zwitterionic dopamine sulfonate are individually exploited as surface and interface modifier to realize the superior water-dispersibility. The surface chemical exchange has been carried out through a ligand exchange process via hydrothermal technique. The exposed iron and gadolinium ions of GdIOPs surface in promoting the T_1_ relaxation are illustrated in Figure 5A, which is attributed to the efficient chemical exchange for protons provided by the surface exposed ions (Zhou et al., 2015b; Shen et al., 2018). *In vivo* MRI performance has been performed at an ultra-high-field of 7.0 T to confirm the efficacy of dual-modal contrast agent served for liver imaging. Figures 5B–E show GdIOPs acting as T_1_-T_2_ dual-modal MRI contrast agent at an ultra-high-field of 7.0 T. MRI T_1_ and T_2_ images have been acquired at the targeted liver, showing both coronal and transverse plane images of liver at preinjection and each certain time postinjection. After 20 min injection, a rapid accumulation of contrast agent nanoparticles is reflected by the brighter contrast in T_1_ imaging and a darker contrast in T_2_ imaging results. Signal to noise ratio changes (ΔSNR) reach to the maximum value for both T_1_ and T_2_ images at 60 min. Subsequently, the values rapidly drop at 240 min. This demonstrates the good metabolism of nanoparticle contrast agent in liver. Consequently, this kind of nanoparticles achieved through surface and interfacial engineering demonstrates its great promising as an ultra-high-field dual-modal MRI contrast agent for accurate diagnosis (Zheng et al., 2016; Kim et al., 2017). Moreover, recent advances of the relaxivity of nanoparticle contrast agents are summarized in Table 1, showing the great potential for ultra-high-field MRI.

**Table 1:** Comparison of the relaxivity of bioresponsive nano-sized contrast agents for ultra-high-field MRI.

**Nanoparticles**	**Size (nm)**	**Magnetic field (T)**	**r_1_ (mM^**−1**^s^**−1**^)**	**r_2_ (mM^**−1**^s^**−1**^)**	**r_2_/r_1_**	**References**
DEG-Gd_2_O_3_	4.6	7.0	4.4	28.9	6.6	Bridot et al., 2007
PEG-Gd_2_O_3_	1.3	11.7	10.4	17.2	1.65	Faucher et al., 2012
MnFe_2_O_4_	7.6	9.4	18.6	227.6	12.2	Kim et al., 2009
CoFe_2_O_4_	8.0	9.4	6.3	392.5	62.3	Kim et al., 2009
PEG-ZnFe_2_O_4_	5.9	9.4	0.60	49	82	Banerjee et al., 2017
Fe_3_O_4_	20	7.0		679		Zhao et al., 2013
Dy_2_O_3_	60/70	17.6/7.0		675/190		Norek et al., 2008
Fe_3_O_4_ sphere	65	7.0		249		Huang et al., 2010
Fe_3_O_4_ brick-like	64.0	7.0	4.3	599	139	Worden et al., 2015
NaDyF_4_ sphere	20.3	9.4		101		Das et al., 2012
PMAO-PEG/ NaDyF_4_	25 × 35	9.4	0.50	204.4	410	Zhang et al., 2016
NaHoF_4_	13.2	7.0	0.35	131.7	376	Ni et al., 2016
PMAO-PEG/NaHoF_4_	17	9.4	0.17	130.6	768	Zhang et al., 2016
QD-CAAKA-DOTA-Dy	5.5	21.1	0.08	57.4	718	Rosenberg et al., 2010
GdIOPs	12	7.0/9.4	6.8/4.3	158.8/167.6	23.4/38.9	Zhou et al., 2015a
NP-PAA-FA	24	7.04/11.7	0.32/0.29	85/146	266/503	Biju et al., 2018

## Bioresponsive Nano-Platforms Mri-Based Multimodal Molecular Imaging

Multimodal imaging is also highly desired to provide complementary information to improve the diagnosis accuracy (Sosnovik et al., 2007; Lee et al., 2012; Li et al., 2016, 2017; Anwaier et al., 2017; Kim et al., 2017). With the emerging the ultra-high-field T_2_ MRI, molecular imaging capable of performing the characterization and measurement of biological process at cellular and molecular levels is highly demonstrated *in vivo* (Shin et al., 2015; Li and Meade, 2019). To realize molecular imaging, specific contrast agent and high sensitive instrument are both required to fulfill the scans with high resolution under high magnetic field (Chen et al., 2014). A variety of MRI contrast agents have been developed till now, which are non-specific contrast agent lacking the ability to reach specific target, targeted contrast agent, smart contrast agent, and the final labeled cells, respectively (Kim et al., 2017; Chang et al., 2020).

Multimodal molecular imaging is tremendously desired in recent years because of the limited resolution and inadequate information provided by each imaging modality. To achieve the high sensitivity and quantitative analysis in clinical diagnosis, multiple imaging modalities with nanoparticle contrast agents can be integrated to make up the complementary information. For instance, MRI possesses the advantages of relative high resolution (25 ~ 100 μm) and superior tissue penetration depth, while its sensitivity requires substantial improvement compared with the direct optical imaging technique. CT has the benefits of high resolution (30 ~ 400 μm) and 3D visual graphing; however, the low sensitivity also becomes the obstacle for its further wide application. Near-infrared fluorescence (NIRF) imaging share the benefit of high sensitivity, while it is constricted by the low spatial resolution. Computed tomography (CT) and MRI can provide the unparalleled structural information, while CT and positron emission tomography (PET) can offer provide the insight into exploring the morphological and functional behaviors. Moreover, other combinations of multimodalities have been also attempted to resolve different upcoming issues in clinics., such as PET/MRI system into the dynamics of brain (Cho et al., 2008, 2013), MRI and CT (Ni et al., 2016), MRI and optical imaging to explore the evolution from single molecule to nanostructures (Harris et al., 2019), triple-modal imaging of NIRF/CT/MRI (Pansare et al., 2012; Hu et al., 2013; Liu et al., 2020), and dual-modality of MRI and single-photon emission computed tomography (SPECT), etc., (Dong et al., 2017; Li et al., 2019).

**Figure 6:**
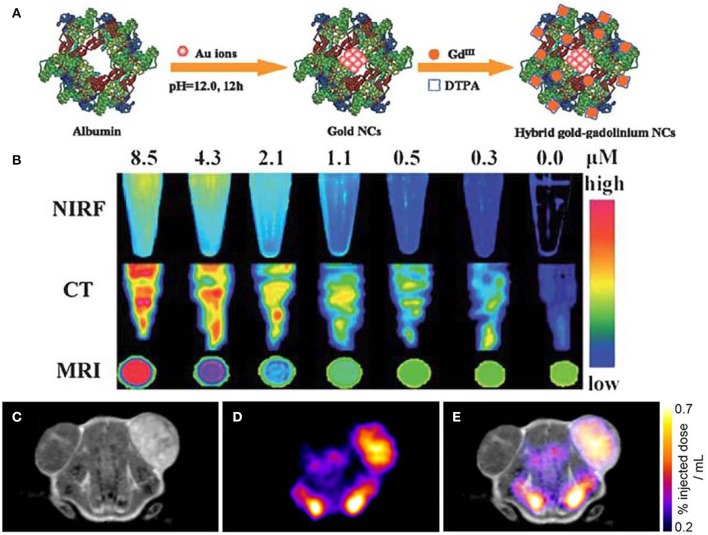
Multimodal molecular imaging: **(A)** schematic procedures of fabricated nanoparticles; **(B)** triple-modal imaging of nanoparticles by using NIRF/CT/MRI (Hu et al., 2013); Copyright 2013, reproduced with permission from The Royal Society of Chemistry. **(C–E)** Dual-modality for *in vivo* imaging via using MRI and SPECT (Patrick et al., 2014); Copyright 2014, reproduced with permission from National Academy of Sciences.

Figure 6A shows the schematic procedures of preparing hybrid nanoparticles. This kind of nanoparticles are used in tumor tissues to perform the diagnosis. The triple-modal NIRF/CT/MRI imaging results demonstrate the efficacy of nanoparticle contrast agent, showing the accuracy of targeting, low body residues. Interestingly, when the content is lowered to 2.1 μM, contrasted signals of NIRF/CT/MRI can be revealed by the contrast agent (Figure 6B). Therefore, the synergistic effect of NIRF/CT/MRI triple-modal imaging has been indicated, showing the complementary information of structural and functional imaging for tumor tissue (Hu et al., 2013). In addition, dual-modality of MRI and SPECT imaging has been endeavored to assist the detection of gene reporters to track viable cells *in vivo*. Figures 6C,D shows the individual MRI image and SPECT image after injecting each specific contrast agent. When MRI and SPECT are integrated for diagnosis, the combined image of dual-modality is shown in Figure 6E, which paves the way for further investigation of cells and gene patterns in gene expression with the multimodal molecular imaging technique (Jung et al., 2020; Song et al., 2020).

On the other hand, nanoparticle functionalization in contrast agent is playing a significant role in monitoring and revealing the diagnosis information at the molecule level, which is accomplished through the combination of dual-modality or tri-modality imaging. Nanoparticle functionalization is performed via indirect or direct surface modification of nanoparticles, where folic acid, oligo nucleotides and peptides, etc., can be exploited as the surfactants (Zhou et al., 2012; Huang et al., 2015; Gao et al., 2017; Thiruppathi et al., 2017). After the functionalization of nanoparticles, these contrast agents are embraced with the advantages of good physical properties, non-invasive, well-dispersed, and homogenous, eventually to achieve the improved efficiency, and abundant modality.

**Figure 7:**
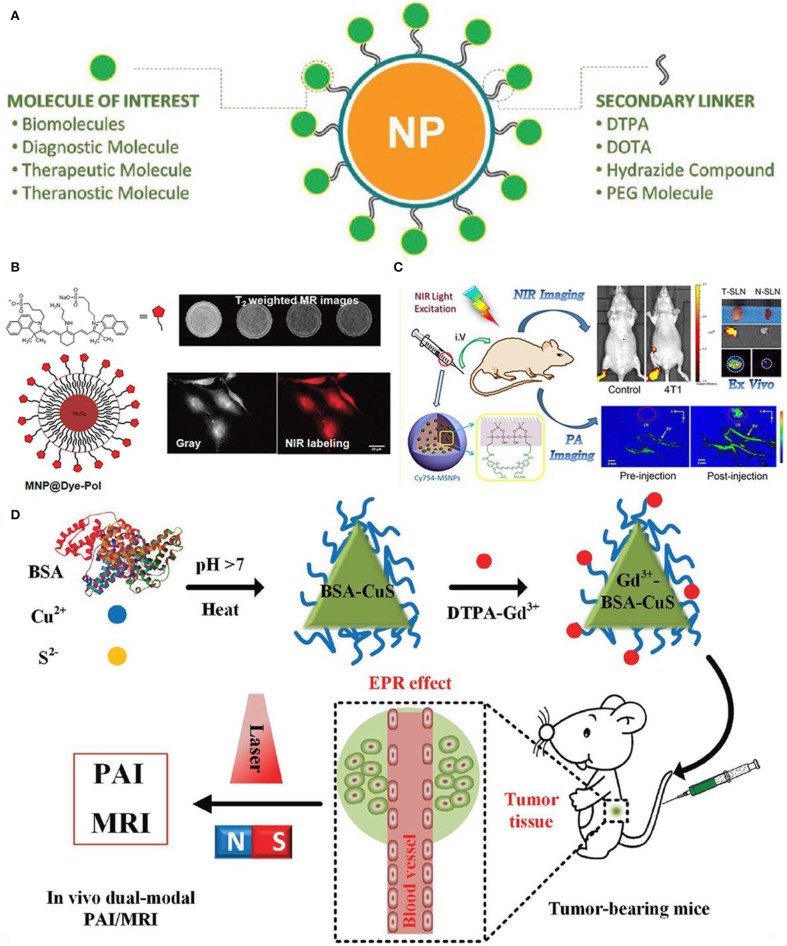
Nanoparticle functionalization enables dual-modal imaging: **(A)** Strategy of linking various diagnosis molecules to nanoparticle agents through a variety of secondary linkers (Thiruppathi et al., 2017); Copyright 2017, reproduced with permission from John Wiley and Sons. **(B)** Magnetic nanoparticle agents functionalized with polymers used for optical and magnetic resonance dual modality imaging (Yen et al., 2013); Copyright 2013, reproduced with permission from American Chemical Society. **(C)** Mapping of sentinel lymph node by photoacoustic (PAI) and near-infrared fluorescent (NIR) (Liu et al., 2015); Copyright 2015, reproduced with permission from American Chemical Society, **(D)** *in vivo* dual-modality of tumor through both PAI and MRI (Gao et al., 2017); Copyright 2017, reproduced with permission from John Wiley and Sons.

Figure 7A shows the functionalized nanoparticles capable of dual-modal imaging, where strategy of linking various diagnosis molecules to nanoparticle contrast agents is illustrated through a variety of secondary linkers (Thiruppathi et al., 2017). To further verify this kind of developed strategy, magnetic nanoparticles are specifically functionalized with polymer linkers and then NIR dye for optical and magnetic resonance imaging (Figure 7B), demonstrating the efficacy of using functionalized nanoparticles for multi-modal imaging (Yen et al., 2013). Moreover, nanoparticles modified with two ligands demonstrate the enhanced targeting efficiency and promote the better clearing of tumor growth at large level, which are ascribed to the enormously increasing uptake of functionalized nanoparticle contrast agents.

In addition, Figure 7C shows the mapping of sentinel lymph node by dual-modality of photoacoustic (PAI) and near-infrared fluorescent (NIR), where fluorescent dye-loaded mesoporous silica nanoparticles imaging contrast are employed to study the tumor metastasis model as well as the underlying molecular level mechanism of dual-modality imaging (Liu et al., 2015). As shown in Figure 7C, surface functionalized nanoparticles in conjunction with dual-modality imaging are used to visualize sentinel lymph nodes up to 2 weeks based on the tumor metastatic model. Moreover, differences of uptake rate and contrast between normal sentinel lymph nodes and metastasized sentinel lymph nodes are compared, showing the feasibility of functionalized nanoparticles in identifying tumor metastasis based on dual-modality imaging results.

Interestingly, water-soluble functionalized nanoparticle contrast agent has been showing its great significance in detecting cancer at early stage. For instance, Figure 7D shows the protein-modified hydrophilic copper sulfide (CuS) used as dual-modality nanoprobe, which opens a new avenue for both photoacoustic imaging and MRI in cancer diagnosis (Gao et al., 2017). This kind of functionalized nanoparticles possess the following advantages: good biocompatible and water-soluble, controllable small size with good stability, feasibly functionalized (Gao et al., 2018). *In vivo* test has been conducted in a subcutaneous tumor mouse with this functionalized nanoparticle contrast agents, showing the improved accuracy in both resolution and contrast. Therefore, with the unique properties of this protein-modified nanostructures, nanoplatform aiming for dual-modality imaging can be designed to target disease diagnosis.

**Figure 8:**
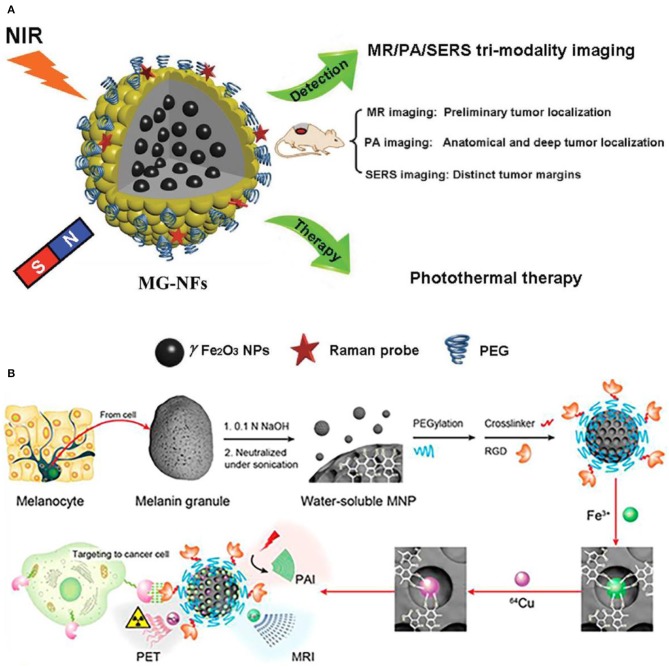
Nanoparticle functionalization enables tri-modality imaging: **(A)** Schematic illustration of surface-enhanced magnetic resonance (MR)/Raman spectroscopy (SERS)/photoacoustic (PA) (MR/SERS/PA) tri-modality imaging of tumor (Huang et al., 2015); Copyright 2015, reproduced with permission from John Wiley and Sons. **(B)** PET/MRI/PAI tri-modality nanoplatform (Fan et al., 2014); Copyright 2014, reproduced with permission from American Chemical Society.

Except for dual-modality imaging, the tri-modality imaging with functionalized nanoparticles has also been endeavored by researchers. Surface-enhanced Raman spectroscopy (SERS) is proposed as a sensitive and non-invasive technique, which provides precise and specific identification of signals in combination with nanoparticle contrast agents. Iterative coating approach is used to reach the rational design and synthesis of core/shell magnetic nanoflower contrast agent (Huang et al., 2015). Thus, remarkable SERS enhancement, superior PA signals, improved relaxivity, and the effective photothermal effect are achieved with this contrast agent. The combination of MRI/PA/SERS techniques has been put forward to achieve the synergistic tri-modality imaging, where MRI is responsible for the contour and localization of tumor diagnosis, PA for deep localizaiton and anatomical and SERS is for margin identification (Figure 8A. In addition, functional biomarker of water-soluble melanin nanoparticle is to target for melanoma imaging (Figure 8B). After conjugating surfactants of α_v_β_3_ integrins and cyclic peptide to melanin nanoparticle, the U87MG tumor accumulation is observed owing to the synergistic effect of enhanced permeability and retention (Fan et al., 2014). With the combinations of different modalities of PET/MRI/PAI techniques, it is anticipated to provide guidance for localizing both superficial and deep tumor surgery. As a result, this kind of water-soluble melanin nanoparticle contrast agent after biomolecules modification shows the tremendous feasibility in multimodal imaging and can be also used as nanoplatform for potential therapeutic applications.

Therefore, through the proof-of-concept design in functionalized nanoparticle contrast agents, a general and versatile strategy can be developed to realize the multimodal imaging with functional molecular probes.

## Targeted Molecular Imaging Agents

Targeted molecular imaging agents in combination with imaging probe are proposed to non-invasively identify cellular processes in varied stages of disease, which generally include metabolic targeted, anti-body targeted, peptide, and activity-based probes (Zhao et al., 2012; Galluzzi et al., 2013; Chen et al., 2014; Craig et al., 2016; Dearling et al., 2016; Lee et al., 2016; Zhang et al., 2017; Mulder et al., 2018; Dammes and Peer, 2020). Peptide shows the advantages of good selectivity and specificity, which belongs to a class of ligand used for MRI (Lee et al., 2010; Craig et al., 2016). Moreover, with the surface modification of targeted molecular imaging agents, the pronounced improvement of targeting efficacy is achieved compared to that of anti-body agents, which is attributed to the controlled size of agent and its large number of ligands (Cai and Chen, 2007). To be eventually exploited as the desired targeted molecular imaging agents, a number of crucial factors need to be considered based on the agent-specific basis principle, such as the toxicity, extent of resection (EOR), the efficient and efficacy of delivery to target issue and the induced side effect. With the targeted imaging agents and probes, studies covering from imaging of breast cancer, cardiovascular disease and neurodegenerative disease has been conducted. However, much remains to be resolved before the practical *in vivo* applications, which includes the aspects of *in vivo* kinetics, efficacy, diagnostic accuracy and sensitivity, biocompatibility, chronic toxicity and the cost.

**Figure 9:**
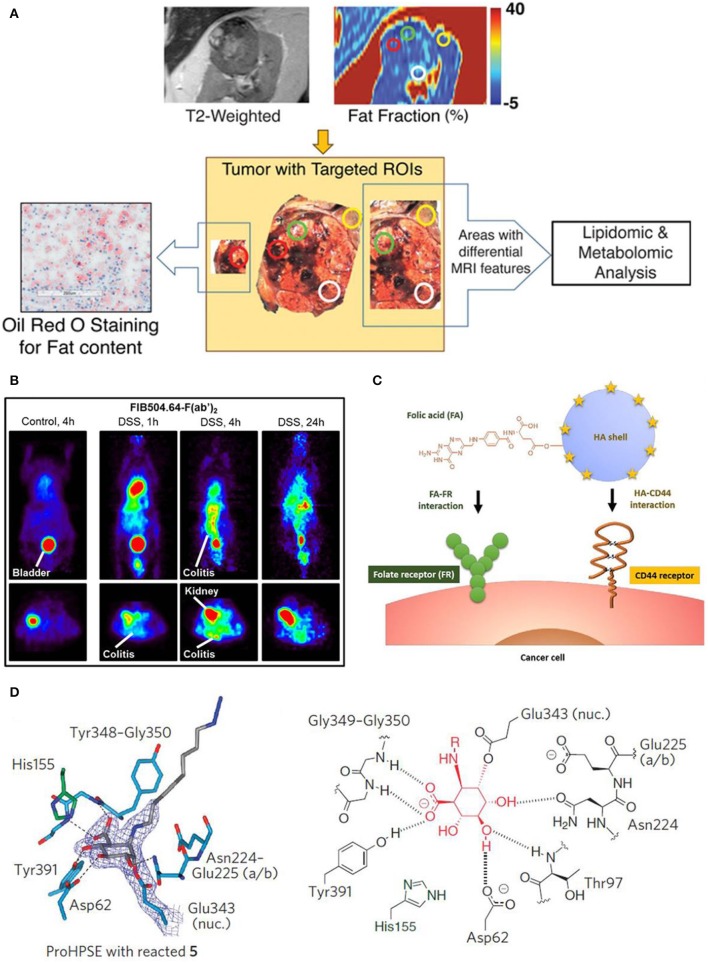
Targeted molecular imaging agents: **(A)** Metabolic targeted agents to proceed the tissue-based analysis with MRI quantitative parameters in anatomical coregistration (Zhang et al., 2017); Copyright 2017, reproduced with permission from Clinical Medicine. **(B)** Anti-body targeted agents (Dearling et al., 2016); Copyright 2016, reproduced with permission from Crohn's & Colitis Foundation of America. **(C)** Schematic diagram of peptide targeted HACE-FA NPs to tumor tissues (Lee et al., 2016); Copyright 2016, reproduced with permission from Elsevier. **(D)** Activity based targeted agents (Zhao et al., 2012); Copyright 2017, reproduced with permission from Nature Chemical Biology.

To predict and assess the metabolic alternation in tumor tissues, a non-invasive quantitative MRI approach has been exploited, where the Dixon-based MRI acts as the biomarker to predict the tumor aggressiveness before surgical intervention. Figure 9A shows the metabolic targeted molecular imaging agents, where tissue-based analysis has been performed with MRI quantitative parameters in anatomical coregistration. After undergoing MRI for patients, the Dixon-based MRI-derived quantitative analysis for *in vivo* fat quantification can be proceeded, showing the role of *in vivo* biomarker of metabolic targeted agents in clear cell renal carcinoma (ccRCC) (Zhang et al., 2017). To evaluate the efficacy of anti-body targeted agents, research work has been conducted to detect the colitis in a mouse model via using the imaging probe. The FIB504.64 shows a pronounced specific uptake, which is revealed by the evident observed colitis (Figure 9B), showing its capability as a promising disease-specific imaging agent (Dearling et al., 2016).

A variety of peptide receptors capable of targeting tumor tissues are reported in molecular biology, such as α_v_β_3_, glucagon-like peptide-1, somatostatin and gastrin-releasing peptide. The strategy of integrating multiple ligands into tumor tissues has been adopted to harvest more potential binding sites and *in vivo* ultrasound intensity. For instance, hyaluronic acid ceramide folic acid nanoparticles (HACE-FA NPs) are reported to target both CD44 and folate receptor (FR) (Figure 9C), showing their synergistic effect to increase affinity of agents to receptors (Lee et al., 2016; Ko et al., 2019). Moreover, to achieve the specific diseased sites imaging, monoclonal antibodies are prevalently used owing to their merits of specificity, affinity and serum stability.

Given the significance of β-glycosidases in human body, a set of β-glycosidase-specific activity-based probes (ABPs) are specifically studied to detect enzymatic activity over a range of glycosidases, demonstrating its role of tracking pathological relevant enzymes and its great potential in discovering the structural and biochemical functionality (Wu et al., 2017). Figure 7D shows the active site interaction with ABPs, where hydrogen bonding interactions identical to that of mature enzyme are illustrated in Figure 9D. Therefore, this activity based targeted agent offers a powerful tool in characterizing enzyme activities.

Upon further improvement of target molecular imaging agents for ultra-high-field MRI, specific molecular therapy with tremendously enhanced accuracy and sensitivity will profoundly impact future clinical diagnosis.

## The Role of Artificial Intelligence for Developing Novel/Bioresponsive Nano Agents for Mri

One of the innovative clinical applications of AI lies in medical imaging, which includes the following aspects: image acquisition, removing the unwanted artifacts, improving the image quality, reducing the contrast agent dose, and shortening the diagnose period (Gong et al., 2018; Shan et al., 2018; Zaharchuk et al., 2018; Codari et al., 2019; Zhu et al., 2019).

**Figure 10:**
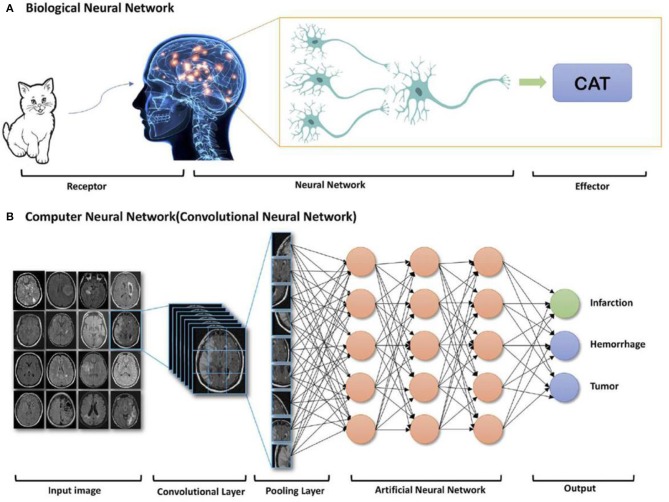
AI deep learning example: components of **(A)** biological neural network and **(B)** computer neural network (Zaharchuk et al., 2018); Copyright 2018, reproduced with permission from American Journal of Neuroradiology.

For biomedical imaging, the image reconstruction can be improved by exploiting machine learning or deep learning of AI, where powerful graphical processing units and neural networks formed in computer will assist the reconstruction processing (Gong et al., 2018; Shan et al., 2018; Zaharchuk et al., 2018). Alternatively, after accessing to large amount of information, the deep learning of AI will be processed and form an algorithm based on these inputs. Figure 10 shows the whole process of biological neural network, where receptor receives a cat as inputting information, after learning process, cat has been reconstructed at the effector side. Moreover, convolutional neural network is another type of learning method of AI, where the algorithm will be configurated to proceed further analysis based on the previous inputs (Bernal et al., 2019). Therefore, deep learning of AI can assist MRI scans to acquire images with enhanced quality.

**Figure 11:**
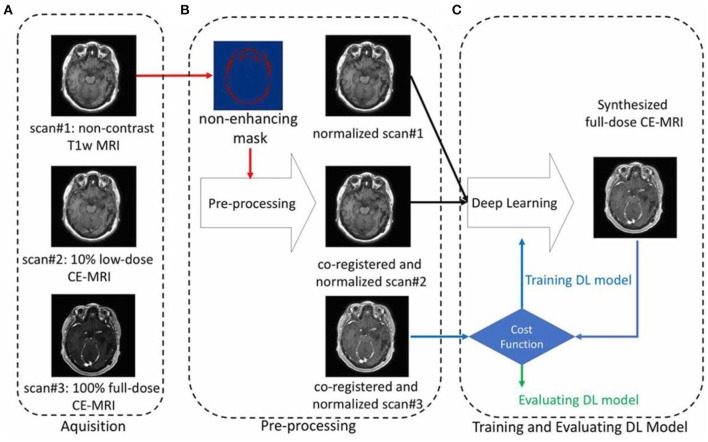
An example showing the contrast enhanced MRI via using the reduced dose of contrast agent through deep learning of AI, including the processes of **(A)** aquisition, **(B)** pre-processing, and **(C)** training and evaluating (Gong et al., 2018); Copyright 2018, reproduced permission from International Society for Magnetic Resonance in Medicine.

To maintain the diagnostic quality, it is also aimed to reduce the MRI contrast agent dose. As heavy metal gadolinium is indispensably used as MRI contrast agent and can remain in human body after scans, researchers are trying to improve the safety of patients after keeping the achieved scan information. Therefore, deep machine learning of AI can be implemented based on designed experiments, that is contrast agent experiment of less dose by comparing with that of no dose and full dose (Gong et al., 2018). For instance, Gong et al. performed an attempt on contrast enhanced MRI with low dose of contrast agent through deep learning of AI (Figure 11). By employing the non-contrast MRI and low-contrast MRI as inputs for deep learning, simulated model/algorithm will be predicted based on the obtained signal difference between non-contrast MRI and low-contrast MRI (Raval et al., 2017). Subsequently, this algorithm will be used to synthesize a full does contrast enhanced MRI to verify the formerly set-up full dose MRI. These results indicate that much useful clinical information can be obtained through using the enormously reduced dose of contrast agent based on the deep learning of AI.

**Figure 12:**
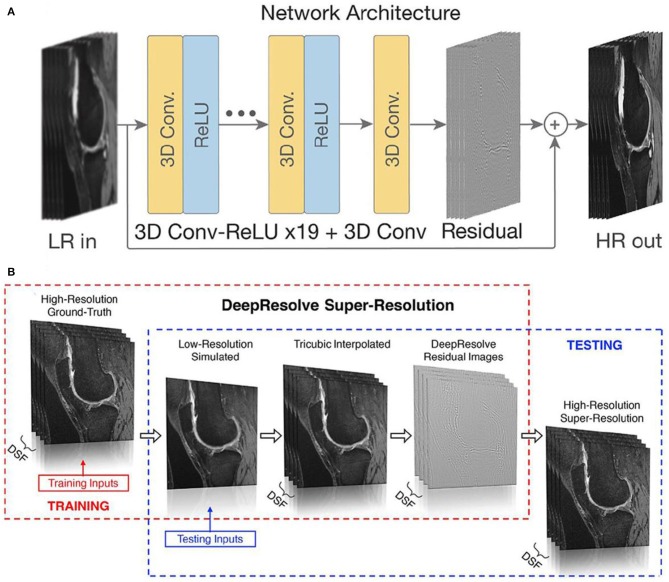
**(A)** An input low-resolution (LR) image computes a high-resolution (HR) image through the deep learning of network; **(B)** schematic diagram of producing high-resolution/super resolution slices (Chaudhari et al., 2018). DSF represents the downsampling factor, where illustrates the ratio between the thickness of ground-truth slice thickness and the downsampled low-resolution slice thickness. Copyright 2018, reproduced with permission from International Society for Magnetic Resonance in Medicine.

On the other hand, the shortened diagnosis time is enormously expected in high field MRI with the aid of AI. To obtain a high or super-resolution MRI, ultra-high magnetic field (over 7 T) is generally required to perform the scan with contrast agent, which will inevitably induce high cost of equipment purchase and high operational cost of long-tern scanning, and the unexpected unsafety issue owing to the high magnetic field. Until now, many attempts have been devoted to exploiting machine learning of AI to replace the high field scan process, where high resolution MRI can be obtained through AI deep learning of low-resolution MRI (Shen et al., 2017a; Mahmud et al., 2018; Wegmayr et al., 2018; Mostapha and Styner, 2019; Nalepa et al., 2020). For instance, Chaudhari et al. developed a super-resolution approach to generate MRI information of thin-slice knee from thicker ones through the configurated convolutional neural networks of AI deep learning (Figure 12) (Chaudhari et al., 2018). Furthermore, Lyu et al. adapted two latest neural network models to realize super-resolution MRI, showing a 2-fold enhancements of resolution in MRI (Lyu et al., 2018). Therefore, with the emerging of AI, it is promising to achieve high resolution MRI without the expensive high field instrument and long-term scanning.

## Prospects and Challenges

**Figure 13:**
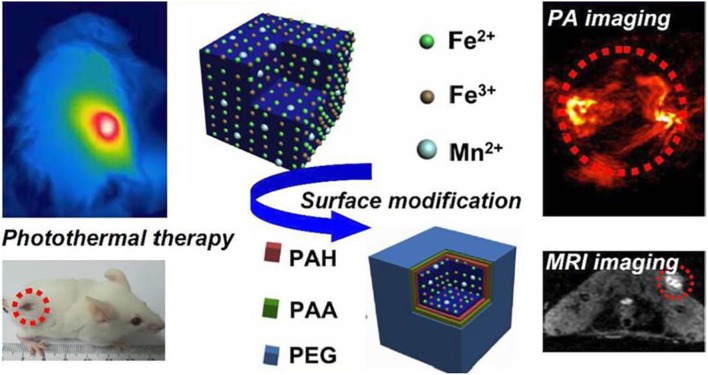
Multimodal probes: from design to biology applications (Zhu et al., 2015); Copyright 2015, reproduced with permission from American Chemical Society.

With the aim of developing contrast agents with good compatibility, biodegradability, high relaxivity for MRI and ultra-high-field MRI, continuous efforts have been devoted to exploring and searching for better contrast agents for MRI. To improve the imaging sensitivity of nanoparticle contrast agents, geometry of size, surface, shape of nanoparticles can be designed, and tuned via surface modification approaches, or other techniques (Zhou et al., 2019). Cutting-edge interdisciplinary subjects of chemistry, physical, biological, and engineering shall also be included to resolve the current limitations of developing nanoparticle contrast agents. After implementing the rational design and surface modification of nanoparticles, this will inevitably promote MRI capable of molecular imaging for better monitoring during biological process (Zhu and Moser, 2017; Basal and Allen, 2018). For instance, Figure 13 shows a representative example about the functionalized nanoparticle contrast agents ranging from the initial design to the final multimodal imaging applications, where doping technique has been adopted to functionalize a variety of nanoparticles with the employed various surfactants, such as poly(allylamine hydrochloride) (PAH), polyacrylic acid, and polyethylene glycol (PEG). Meanwhile, nanoparticle contrast agents are not just limited to Fe^2+^, Fe^3+^, Mn^2+^ based nanoparticles (Zhu et al., 2015). Other bioresponsive nano-sized contrast agents used for high field magnetic resonance imaging can also be employed for the surface functionalization, eventually to act as the molecular probes for multimodal imaging of photoacoustic (PA) imaging and MRI imaging, etc. (He et al., 2018). Consequently, wide applications of high field MRI, multimodal imaging, and theragnostic technique into MRI will be also promoted by the synergistic effect of both molecular probes and a variety of imaging techniques.

**Figure 14:**
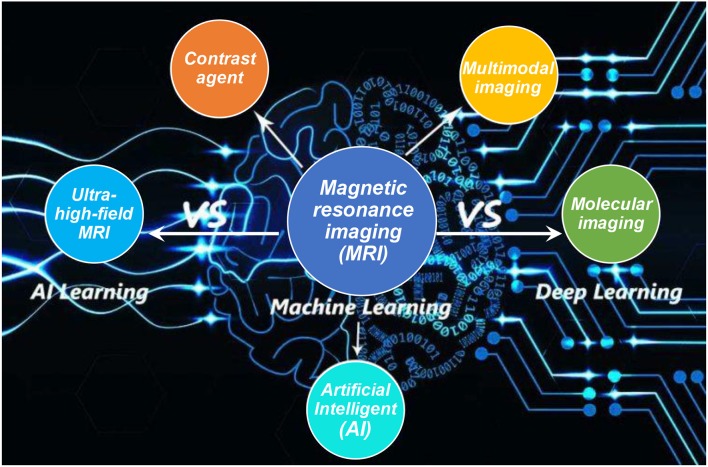
MRI and AI to varies of imaging development.

In addition, based on the design principles of nanoparticle contrast agents, crucial aspects in terms of easy-processable and low cost are considered to meet the large-scale demands, which can be resolved with the assistance of AI technology. As the fabrications of nanoparticle contrast agents focus on optimizing a variety of parameters, with the emerging of AI, the algorithm based on the deep learning will complete all these tasks prior to perform the final optimized experiments for verification, which will save substantial time, efforts and achieve the high resolution MRI (Moser et al., 2017; Raval et al., 2017). Furthermore, for future MRI, it is suggested to employ the convolutional neural networks in AI deep learning to get the multimodal imaging information with high resolution (Figure 14), where the conventional MRI can only achieve single information with the applied high magnetic field (over 7 T) (Donatelli et al., 2018; Henning, 2018; Turing, 2019).

Molecular imaging enables the quantitative characterization and measurement of biological diagnosis at the cellular and molecular level, which will inevitably advance the modern and future medical imaging and diagnosis (Wu and Shu, 2018; Li and Meade, 2019). However, the current critical issue lies in developing novel and appropriate contrast agents to meet the biological compatibility for different species (Basal et al., 2017). MRI with nanoparticle contrast agent offers one desired solution to realize molecular imaging. However, the poor sensitivity of MRI limits its wide application in clinics. Efforts can be made toward integrating the varied modalities/strengths of different instruments to avoid the problems existed in an individual instrument. Alternatively, interdisciplinary cooperation among different subjects should be strengthened to resolve the limited resolution of imaging, because molecular imaging requires the joint research of radiology, materials science and ultrasonic medicine.

To sum it up, multimodal molecular imaging agents and specific targeted molecular imaging agent will be both expected in the molecular imaging in future clinic application. With the aid of AI, a major breakthrough can be expected in multimodal molecular imaging for molecular biology clinics, despite molecular imaging combined with AI learning is still at the initial stage.

## Author Contributions

The author confirms being the sole contributor of this work and has approved it for publication.

### Conflict of Interest

The author declares that the research was conducted in the absence of any commercial or financial relationships that could be construed as a potential conflict of interest.
